# Drug–Polymer Interactions in Acetaminophen/Hydroxypropylmethylcellulose Acetyl Succinate Amorphous Solid Dispersions Revealed by Multidimensional Multinuclear Solid-State NMR Spectroscopy

**DOI:** 10.1021/acs.molpharmaceut.1c00427

**Published:** 2021-08-10

**Authors:** Andrea Pugliese, Michael Toresco, Daniel McNamara, Dinu Iuga, Anuji Abraham, Michael Tobyn, Lucy E. Hawarden, Frédéric Blanc

**Affiliations:** † Department of Chemistry, University of Liverpool, Crown Street, Liverpool L69 7ZD, United Kingdom; ‡ Chemical Engineering Department, Rowan College of Engineering, Rowan University, Mullica Hill Road, Glassboro, New Jersey 08028, United States; § Drug Product Development, Bristol-Myers Squibb, One Squibb Drive, New Brunswick, New Jersey 08903, United States; ∥ Department of Physics, University of Warwick, Gibbet Hill Road, Coventry CV4 7AL, United Kingdom; ⊥ Drug Product Development, Bristol-Myers Squibb, Reeds Lane, Moreton CH46 1QW, United Kingdom; # Stephenson Institute for Renewable Energy, University of Liverpool, Peach Street, Liverpool L69 7ZF, United Kingdom

**Keywords:** acetaminophen (paracetamol), hydroxypropylmethylcellulose acetyl succinate (HPMC-AS), amorphous solid dispersion, solid-state NMR, drug−polymer interactions, multidimensional NMR

## Abstract

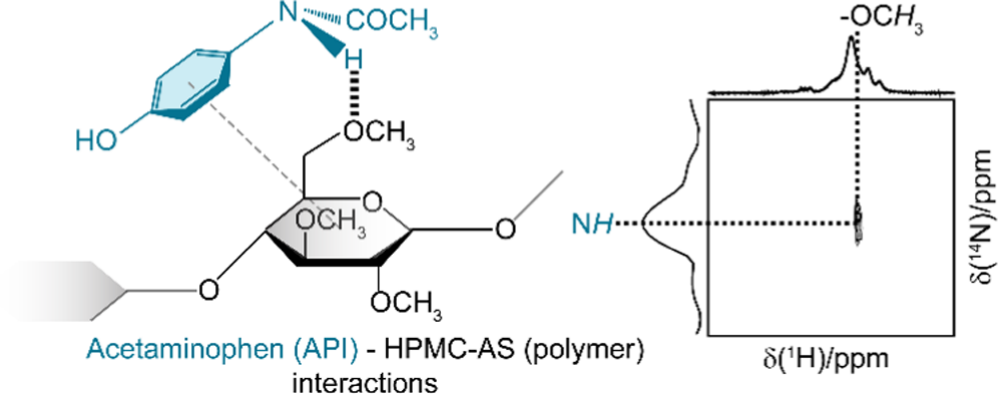

The bioavailability of insoluble crystalline active pharmaceutical ingredients (APIs) can be enhanced by formulation as amorphous solid dispersions (ASDs). One of the key factors of ASD stabilization is the formation of drug–polymer interactions at the molecular level. Here, we used a range of multidimensional and multinuclear nuclear magnetic resonance (NMR) experiments to identify these interactions in amorphous acetaminophen (paracetamol)/hydroxypropylmethylcellulose acetyl succinate (HPMC-AS) ASDs at various drug loadings. At low drug loading (<20 wt %), we showed that ^1^H–^13^C through-space heteronuclear correlation experiments identify proximity between aromatic protons in acetaminophen with cellulose backbone protons in HPMC-AS. We also show that ^14^N–^1^H heteronuclear multiple quantum coherence (HMQC) experiments are a powerful approach in probing spatial interactions in amorphous materials and establish the presence of hydrogen bonds (H-bond) between the amide nitrogen of acetaminophen with the cellulose ring methyl protons in these ASDs. In contrast, at higher drug loading (40 wt %), no acetaminophen/HPMC-AS spatial proximity was identified and domains of recrystallization of amorphous acetaminophen into its crystalline form I, the most thermodynamically stable polymorph, and form II are identified. These results provide atomic scale understanding of the interactions in the acetaminophen/HPMC-AS ASD occurring via H-bond interactions.

## 1 Introduction

Biopharmaceutical class II active pharmaceutical ingredients (APIs) (or drugs) exhibit poor bioavailability as a result of low aqueous solubility, accompanied by high biological membrane permeability.^1^ API can exist either in the crystalline form, characterized by a three-dimensional (3D) structure in which molecules are packed in a regularly ordered repeating pattern, or amorphous form defined as an ensemble of molecules/units arranged randomly. The energy barrier required to break down the long-range structure means that crystalline systems can show low solubility and a low kinetic rate of dissolution. In amorphous systems, the lack of long-range order greatly enhances the apparent solubility and rate of dissolution.^2^ From a thermodynamic viewpoint, the crystalline state is low energy and stable in contrast to the amorphous state, which is marked as high energy and unstable. The metastable nature of the amorphous state leads to the likelihood of physical instability and recrystallization promoted by external factors such as temperature or humidity.^3^ Converting crystalline drugs to their amorphous counterpart is one of the most promising approaches in pharmaceutical material sciences to enhance APIs’ solubility and bioavailability. This strategy can be adopted only as long as a supersaturated solution of amorphous API can be maintained in the aqueous medium over time.^4^

Amorphous solid dispersions (ASDs) have been extensively used to stabilize supersaturated solution of APIs, resulting in a general increase for oral bioavailability of poorly soluble drugs.^5−7^ An ASD can be defined as a dispersion of one or more APIs in a solid-state inert carrier, usually an amorphous polymer,^8^ and can be prepared by a range of manufacturing processes,^9^ including spray drying,^10^ spray freeze drying,^11^ and hot melt extrusion.^12^ Polymers such as poly(ethylene glycol) (PEG),^13^ poly(ethylene oxide) (PEO),^14^ poly(vinylpyrrolidone) (PVP),^15^ poly(vinylpyrrolidone)–poly(vinyl acetate) (PVP–VA),^16^ hydroxypropylmethylcellulose (HMPC),^17^ and hydroxypropylmethylcellulose acetyl succinate (HPMC-AS)^18^ have been successfully used in ASDs. In particular, HPMC-AS has recently been suggested as a promising solid matrix to formulate ASDs^19^ due to its high glass transition temperature, Tg, in the order of 120 °C,^20^ its amphiphilic nature arising from the existence of hydrophilic (e.g., acetyl, A) and hydrophobic (e.g., succinoyl, S) functional groups, and the capability to tune the A and S contents.

It has been demonstrated that the polymer in ASDs plays a crucial role in stabilizing the amorphous form of the drug.^21^ The choice of a suitable polymer to formulate a specific dispersion largely depends on several chemical–physical properties such as Tg, thermal stability, dissolution profile, performance in dissolving API, and capability to stabilize amorphous drugs.^22^ These characteristics contribute to the stabilization of the ASD, which is due to the polymer’s antiplasticizing effect, reducing molecular mobility of the amorphous API, and the formation of specific API–polymer interactions.^21^ Intermolecular interactions such as hydrogen bonding (H-bond), ionic forces, π–π, or electrostatic interactions are well established as the most significant interactions capable of stabilizing such dispersed systems^4^ by inhibiting recrystallization phenomena in the amorphous matrix and preventing competitive API–API or polymer–polymer intramolecular interactions. Recently, the HPMC-AS polymer^23^ has been widely used to prepare ASD due to its remarkable ability in stabilizing amorphous dispersions arising from the formation of strong API–HPMC-AS interactions.^24^

The elucidation of the nature of the interaction between drugs and polymers and detecting recrystallized drugs in ASDs constitute some of the most significant challenges in pharmaceutical material sciences and require the exploitation of a range of characterization approaches, often combining powder X-ray diffraction (PXRD), thermal analysis, vibrational methods, and solid-state nuclear magnetic resonance (NMR) spectroscopy.^25,26^ The PXRD patterns of amorphous solids result in broad diffuse scattering signals due to the lack of long-range order. Nevertheless, PXRD methods can provide significantly useful information on the residual crystalline content in ASDs, for example, during stability studies.^27^ Thermal analysis, including differential scanning calorimetry (DSC) and temperature-modulated DSC (mDSC),^28^ have been employed to estimate the residual crystallinity in amorphous systems^29^ and are often used to determine Tg values and detect thermal events revealing crystallization and melting phenomena.^30^ DSC therefore allows to detect miscibility of individual components of an ASD, where the observation of a single Tg indicates miscibility between API and the polymer.^31^ Gordon–Taylor’s (GT) model^32^ can be used to estimate the Tg of an ideal binary mixture (Tg_mix_) with significant deviations between predicted Tg_mix_ and experimentally determined Tg providing useful information about the interactions between the components in the mixture,^4,25,26^ as the presence of API–API or API–polymer interactions can affect the Tg value of the system, while agreement suggests systems with the absence of specific drug–polymer interactions. Furthermore, access to ASD stability can be also obtained using thermodynamic modeling and short- to medium-term physical stabilities of several API–polymer blend ASDs. These include acetaminophen–HPMC-AS dispersions under controlled temperature and relative humidity (RH) conditions which have been determined for the 20 and 40 wt % formulation to be up to 6 and 1 month(s), respectively.^33^ This work highlights a reduction of stability of these systems with the increase of the polymer content and an increase of RH.

A range of analytical methods including vibrational, Raman, Fourier-transform infrared (FT-IR) spectroscopy, and solid-state nuclear magnetic resonance (NMR) spectroscopies have been used to provide atomic scale information about solid dispersions. Raman applications include the measurements of crystallization rate^34^ and mapping solid dispersions to identify and discriminate crystalline/amorphous domains.^35^ FT-IR methods can be used to probe H-bonds for specific functional groups including hydroxyl, amino, and carbonyl groups when present in the API and/or the polymer molecular structure.^36^ It has been demonstrated that when those functional groups are involved in H-bonding interactions, a simultaneous decrease in the stretching frequency and a widening of their absorption bands are observed due to smaller intermolecular distances between the donor–acceptor groups.^37^

NMR spectroscopy has proved itself as a powerful technique by providing an invaluable source of both structural and dynamics information at the atomic scale thereby being demonstrated as one of the most powerful methods of characterization. In particular, in the field of pharmaceutical sciences,^38,39^ NMR allows the determination of the structure of drugs^40^ and polymers.^41^ Recently, NMR has emerged as a robust approach in (pharmaceutical) amorphous dispersions to identity site-specific API–polymer intermolecular interactions from changes in chemical shift values.^42−45^ For example, using one-dimensional (1D) and two-dimensional (2D) NMR experiments, electrostatic interactions and H-bonding were identified in amorphous posaconazole (POSA) dispersion in HPMC-AS and involved the POSA’s triazole and difluorophenyl ring moieties with some of the HPMC-AS’s substituent groups.^44^ The presence of π–π aromatic packing interaction between POSA and HPMC-phthalate (HPMC-P) amorphous dispersion has also been highlighted.^44^ Drug–polymer interactions in carbamazepine (CBZ) in HPMC, HPMC-A, and HPMC-S dispersions have also been established^42^ and identified H-bonding between the CBZ’s −NH_2_ group with the acetyl moiety in HPMC-A and between both CBZ −NH_2_’s and carbonyl groups of the succinyl group in HPMC-S. This demonstrates the important role that both acetyl and succinyl groups of HPMC-AS could play in the formation of stable API–polymer connections. 2D NMR techniques that include homonuclear and heteronuclear correlation spectroscopy are widely used to detect intramolecular interaction by exploiting the homo- and heteronuclear through-space dipolar coupling between the nuclei. To increase the NMR sensitivity and hence to have access to high-resolution spectra and enabling proton detection, the use of ultrafast magic angle spinning (MAS) experiments, with frequency in the 50–110 kHz range, has also recently emerged. They enable fast characterization of pharmaceutical compounds and formulation by probing API–polymer interaction,^45^ allowing NMR crystallography approaches^46^ and understanding of low drug-loaded formulation.^47^ The ^14^N–^1^H heteronuclear multiple-quantum coherence (HMQC)^48^ experiment carried out at the high magnetic field and at ultrafast MAS conditions under direct ^1^H signal detection has been robustly employed to probe interactions in crystalline systems^49,50^ and recently to highlight molecular association and interactions in amorphous dispersions.^51,52^^14^N–^1^H HMQC spectra were used to identify hydrogen bonding interaction in a nicotinamide palmitic acid cocrystal and acetaminophen–PVP amorphous dispersion.^50^ The versatility of this experiment was demonstrated by providing information on the symmetry of the nitrogen environment and through-space proximities in paclitaxel-loaded polymer micelles amorphous formulations.^52^ The ^14^N–^1^H HMQC experiment has however, to the best of our knowledge, not been used so far to investigate API–polymer interactions in HPMC-AS-based amorphous formulations.

**Figure 1:**
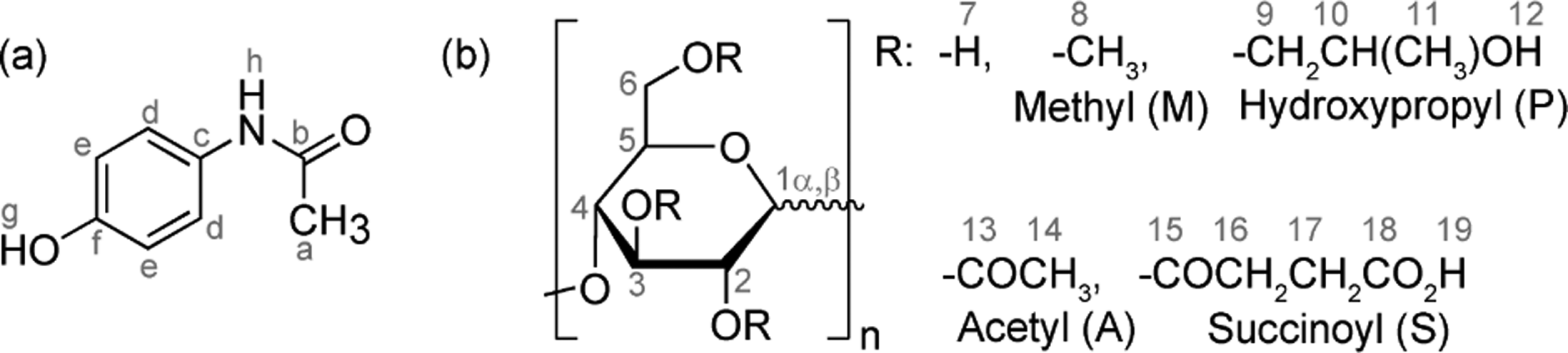
Chemical structure of (a) acetaminophen and (b) HPMC-AS polymer. HPMC-AS consists of a cellulose ring bonded with various R groups that include hydrogen, methyl (M), hydroxypropyl (P), acetyl (A), and succinoyl (S) groups. The wavy bond in the cellulose ring indicates that the cellulose ring can exist in two different cyclic hemiacetal configurations, called α- and β-glucopyranose, distinguishable from the different configurations of the anomeric carbon C_1_. The lettering and numbering are used for all NMR spectral assignments throughout.

Here, we report the stability of amorphous acetaminophen in HPMC-AS ASDs at different drug loadings by identifying the presence of drug–polymer intramolecular interactions with multinuclear multidimensional NMR experiments. Acetaminophen (Figures 1 and SI-1) is one of the most widely used API and its chemical–physical data, including melting point and solubility profiles, as well as crystalline data,^53,54^ NMR spectra,^55^ are largely known. The HPMC-AS polymer was chosen as excipient due to its excellent capacity to stabilize amorphous dispersion.^42,44^ Morevoer, the lack of overlap between acetaminophen and polymer signals in the ^13^C NMR spectra allows monitoring of the changes in chemical shift and line width of the signals of both components to establish API–polymer interactions and crystalline/amorphous behavior. Multidimensional multinuclear MAS NMR data enable access to structural information in the solid state, highlighting the presence of API–polymer intermolecular interactions for ASDs with drug loading <20 wt % and providing useful indications of their stability. The approach also suggests the absence of API–polymer intermolecular interaction in the 40 wt % ASD and rather identifies signals corresponding to crystalline acetaminophen interacting with itself.

## 2 Experimental Section

### 2.1 Materials

ASDs were prepared using acetaminophen form I (99.5%) purchased from Spectrum Chemical Company and HPMC-AS polymer M grade obtained from Shin-Etsu Chemical Co. (lot # 6033060, M content = 23.4%, P content = 7.3%, A content = 8.8%, and S content = 11.2%). Sigma-Aldrich’s acetaminophen form I was used to carry out the PXRD analysis. The dipeptide β-AspAla was obtained from Bachem. All materials were used as received.

### 2.2 Synthesis of ASDs

General procedure of the preparation of ASDs: Gram-scale batches formulated at 10, 20, and 40 wt % of acetaminophen were manufactured using a custom-built small-scale spray dryer. Spray dry solution of acetaminophen and the polymer containing 2.5% solid (acetaminophen and HPMC-AS) were sprayed at 65–70 °C from acetone (80 mL) using heated nitrogen gas through a two-fluid spraying nozzle (2050 LC/64AC, Spraying Systems Co.). The ASD was then collected by filtration from the spray dryer and dried overnight in vacuo. ASDs were stored in a freezer kept at low temperature (−80 °C) to prevent API recrystallization.

Synthesis of 10 wt % acetaminophen in HPMC-AS ASD: This formulation was prepared according to the general procedure highlighted above using acetaminophen (0.2 g, 1.3 mmol) and HPMC-AS (1.7 g).

Synthesis of 20 wt % acetaminophen in HPMC-AS ASD: This formulation was prepared according to the general procedure highlighted above using acetaminophen (0.4 g, 2.7 mmol) and HPMC-AS (1.6 g).

Synthesis of 40 wt % acetaminophen in HPMC-AS ASD: This formulation was prepared according to the general procedure highlighted above using acetaminophen (0.8 g. 5.3 mmol) and HPMC-AS (1.2 g).

### 2.3 PXRD Measurements

Laboratory PXRD data were collected using a PANalytical Empyrean diffractometer equipped with a high throughput transmission geometry, focusing mirror, 1/2° divergence, and antiscatter slits, 4 mm beam mask, 0.04° soller slits, with Cu Kα of 1.541874 Å. PXRD patterns were measured over the 2θ range 2–40° over 1 h.

### 2.4 Standard and Modulated DSC Measurements

DSC experiments were performed using a DSC Q1000 (TA Instruments, DE) system using TA-Tzero aluminum pans loaded with an amount of around 10 mg of the sample. Standard DSC analyses were carried out using a cool–heat–cool cycle method in which the sample was cooled to −15 °C and heated up to 160 °C with a ramp of 10 °C min^–1^, and then, after an isotherm of 5 min, a cool ramp of 20 °C min^–1^ was applied back down to −15 °C. mDSC experiments were carried out using a heating ramp of 2.5 °C min^–1^ with a modulation amplitude of 1.5 °C every 60 s.

### 2.5 Solid-State NMR Experiments

^1^H NMR spectra were recorded on a Bruker 800 MHz (18.8 T) Avance Neo NMR spectrometer using a Bruker 1.3 mm HX MAS probe or on a Bruker 850 MHz (20 T) Avance Neo spectrometer equipped with a Bruker 1.3 mm triple-resonance HXY MAS probe in the double resonance (DR) mode. All spectra were recorded under a MAS frequency of ν_r_ = 60 kHz. ^1^H pulses were carried out at a radio frequency (rf) field amplitude of 100 kHz. ASDs ^1^H spin-lattice relaxation times *T*_1_ were recorded at 18.8 T from saturation recovery experiments and fitted to a stretch exponential function of the form 1 – exp[−(τ/*T*_1_)^α^] in which τ is the variable delay and α the stretch factor ranging from 0.5 and 1.

All ^13^C/^15^N cross-polarization (CP) and two-dimensional (2D) ^1^H–^13^C/^15^N CP heteronuclear correlation (HECTOR) experiments were performed on a Bruker 400 MHz (9.4 T) Avance III HD NMR spectrometer equipped with a Bruker 4 mm triple-resonance HXY MAS probe in the DR mode tuned to ^1^H and ^13^C or ^15^N at Larmor frequencies of 400.1, 100.6, and 40.5 MHz. ^1^H pulses and SPINAL-64 heteronuclear decoupling^56^ during ^13^C/^15^N detection were carried out with an rf field amplitude of 83 kHz. All experiments were performed under a MAS frequency of ν_r_ = 12.5 kHz for ^13^C and 10 kHz for ^15^N and using a recycle delay of 1.3 × ^1^H *T*_1_s obtained as above (data at 9.4 T, not given). The Hartmann–Hahn^57^ conditions for ^13^C CP were achieved using a ^13^C rf amplitude of around 45 kHz ramped to obtain maximum signal at a ^1^H rf field of 60 kHz, and for ^15^N CP, a ^15^N rf amplitude of 28 kHz ramped to obtain maximum signal at a ^1^H rf field of 50 kHz was used. A 2 ms contact time during ^13^C CP and optimized CP contact times of 1 ms for the amorphous material and 6 ms for the crystalline sample during ^15^N CP were used. ASD’s ^1^H spin-lattice relaxation times in the rotating frame (*T*_1ρ_) were obtained at 9.4 T, using a spin-lock pulse sequence through ^13^C detection via CP, at ^1^H frequencies of ω_1_/2π of 40 and 83 kHz and fitted to a stretch exponential function of the form exp[−(τ/*T*_1ρ_)^β^] (with β ranging between 0.2 and 1). ^13^C *T*_1_s were obtained at 9.4 T from ^13^C inversion recovery via CP experiments and fitted to an expression of the form exp[−(τ/*T*_1_)^γ^] (with γ ranging from 0.4 to 1). Frequency switched Lee–Goldberg (FSLG) homonuclear decoupling^58^ during the ^1^H *t*_1_ evolution time in the 2D CP HETCOR spectra was obtained at an rf amplitude of 83 kHz and an offset of 60 kHz. Experimentally determined ^1^H scaling factors λ_exp_ for FSLG (as measured on l-alanine using the experimental conditions given above) were used to recover the full ^1^H chemical shifts δ(^1^H)^MAS^ from the scaled-down apparent chemical shifts δ(^1^H)^APP^ according to δ(^1^H)^APP^ = λ_exp_δ(^1^H)^MAS^ that result from this decoupling.

^14^N–^1^H HMQC experiments were carried out using a Bruker 800 MHz (18.8 T) Avance Neo NMR spectrometer equipped with a Bruker 1.3 mm HX MAS probe tuned to ^1^H and ^14^N at 800.3 and 58.7 MHz, respectively, or using a Bruker 850 MHz (20.0 T) Avance Neo NMR spectrometer equipped with a Bruker 1.3 mm triple-resonance HXY MAS probe operating in the DR mode tuned to ^1^H and ^14^N at 850.2 and 61.4 MHz, respectively. Experiments were performed under a MAS frequency ν_r_ = 60 kHz. In the ^14^N–^1^H HMQC pulse sequence used, heteronuclear dipolar couplings were reintroduced via rotary resonance recoupling, *R*^3^,^59^ on the *n* = 2 resonance condition,^48^ using an *x*, −*x* phase inversion^60^ of individual block lengths of one rotor period of 16.7 μs at an rf amplitude of 120 kHz (2 × MAS frequency). ^1^H and ^14^N pulses were performed at an rf amplitude of 100 and 72 kHz, respectively. HMQC spectra were processed after removal of the first few points in the free induction decay (FID) using a home-built macro running on TopSpin to reduce baseline distortion and residual *t*_1_ noise from the spectrum.

^1^H, ^13^C, and ^15^N spectra were externally referenced to the NH proton of the dipeptide β-AspAla at 8.0 ppm,^50^ the tertiary carbon of adamantane at 29.45 ppm,^61^ and to glycine at −347.2 ppm,^62^ respectively. ^14^N shifts were referenced to solid NH_4_Cl at −341.3 ppm,^62^ which has a cubic ^14^N site.^63^ Magic angle calibrations were achieved by maximizing either the separation of NH_3_ and NH resonances of the dipeptide β-AspAla in the ^14^N–^1^H HMQC spectrum or the number of rotational resonances in the time domain of the ^79^Br FID of KBr. The errors associated with ^1^H, ^13^C, and ^15^N chemical shifts and ^14^N parameters are given in the respective tables. ^1^H *T*_1ρ_ and ^13^C *T*_1_ fitting data were carried out using MATLAB R2017a. Deconvolution of the experimental spectra was carried out in TopSpin 4.0.5 using the solid line shape analysis routine.

## 3 Results and Discussion

### 3.1 PXRD Characterization

**Figure 2:**
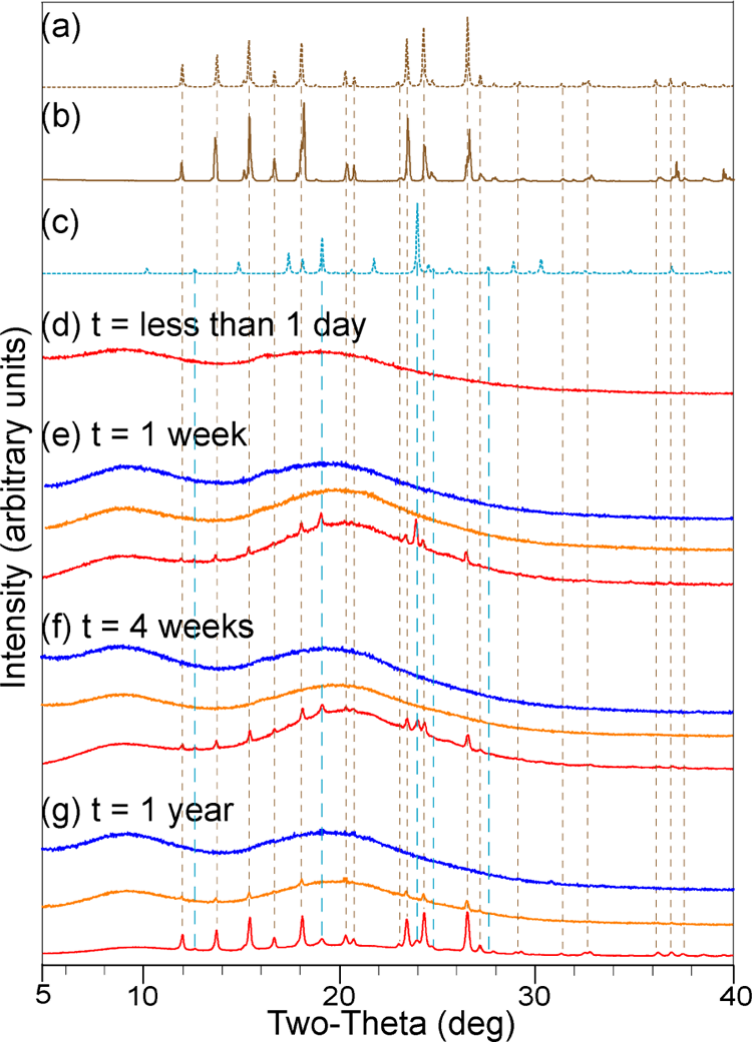
PXRD patterns of (a) simulated acetaminophen form I from CSD (refcode HXACAN01, brown dotted lines),^64^ (b) experimental acetaminophen form I (brown full lines), and (c) simulated acetaminophen form II from CSD (refcode HXACAN23, light blue dotted lines).^64^ Comparison of PXRD patterns of 10 wt % (dark blue), 20 wt % (orange), and 40 wt % (red) for the acetaminophen–HPMC-AS ASDs at times of (d) less than 1 day, (e) 1 week, (f) 4 weeks, and (g) 1 year at RT (around 20 °C) and ambient RH (ranging from 30 to 50%). After 1 year, the 10 wt % ASD still shows an amorphous state, while in the 20 wt % traces of recrystallization to acetaminophen I is observed and further confirmed by the ^13^C CP HETCOR spectra (Figure SI-2). In 40 wt % ASD, acetaminophen forms I and II are detected after only 1 week.

Acetaminophen exists in three polymorphic forms:^53,64,65^ monoclinic form I (space group *P*2_1_/*a* and the number of asymmetric units in the cell, *Z*′ = 1), which is the most thermodynamically stable form; orthorhombic form II (space group *Pcab*, *Z*′ = 1) polymorph, and a highly metastable form III (space group *Pca*2_1_, *Z*′ = 2). Time-dependent PXRD pattern measurements (Figure 2) on acetaminophen–HPMC-AS ASDs at 10 wt % (in dark blue), 20 wt % (in orange), and 40 wt % (in red) loadings were carried out over a 1-year period of exposure at room temperature (RT, around 20 °C) and ambient relative humidity (RH, ranging from 30 to 50%) to monitor the chemical stability of the systems and potential recrystallization phenomena. The diffraction patterns of the 10 wt % (in dark blue) ASD exhibit the typical broad signal of an amorphous material and the absence of Bragg peaks up to 1 year, indicating a strong tendency of this system to remain in the amorphous state. The 20 wt % ASD shows a typical broad signal of an amorphous material only up to 4 weeks at RT and ambient RH after which reflections from acetaminophen form I start to appear. This is in sharp contrast with the PXRD data in 40 wt % ASD that shows recrystallization after only 1 week and, interestingly, to a mixture of both acetaminophen form I and II polymorphs.

### 3.2 Thermal Characterization

The GT model was used to estimate the predicted Tg_mix_ values of the acetaminophen–HPMC-AS dispersion at different drug loading wt % from the following expression
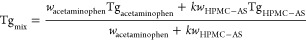
1where w and Tg are the weight fractions and glass transition temperature of each component, respectively, and *k* a constant related to the density ρ (ρ_acetaminophen_ = 1.29 g cm^–3^, ρ_HPMC-AS_ = 1.28 g cm^–3^)^33^ and given by
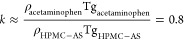
2

**Figure 3:**
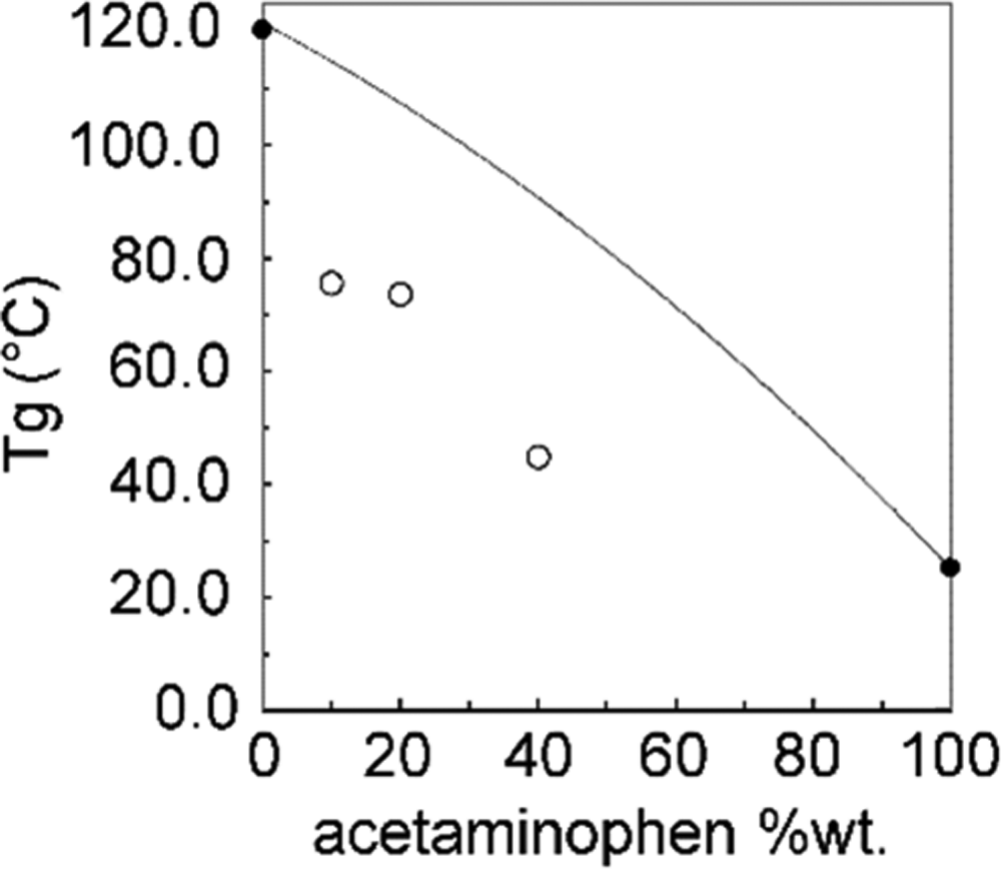
Tg values of acetaminophen–HPMC-AS dispersions obtained at different acetaminophen loadings in HPMC-AS polymer-based ASDs. Experimentally obtained values for the ASDs, individual component, and predicted values from the GT model based on eq 1 are given in empty circles (**○**), filled circles (●), and solid line, respectively.

Supporting information Table SI-1 summarizes Tgs for the individual components as well as the predicted and experimental Tg_mix_ values obtained for the 10, 20, and 40 wt % ASDs with negative deviations from predicted Tgs represented in Figure 3. This demonstrates the nonideal drug–polymer mixture,^7^ the negative deviations suggesting that intramolecular interactions between like species (drug–drug or polymer–polymer) dominate. However, importantly, this does not exclude the presence, to a lesser extent, of intramolecular drug–polymer interactions (see below).^66^ Additionally, negative deviations can also be interpreted as being indicative of nonideal additivity of volume for the two components and points out of a likelihood of phase separation of the system.^67^ The largest deviation is found for the 40 wt % solid dispersion and suggests that at, among the ASDs studied, recrystallization phenomena and phase separation occur more quickly in this formulation.

### 3.3 Solid-State NMR Data

**Figure 4:**
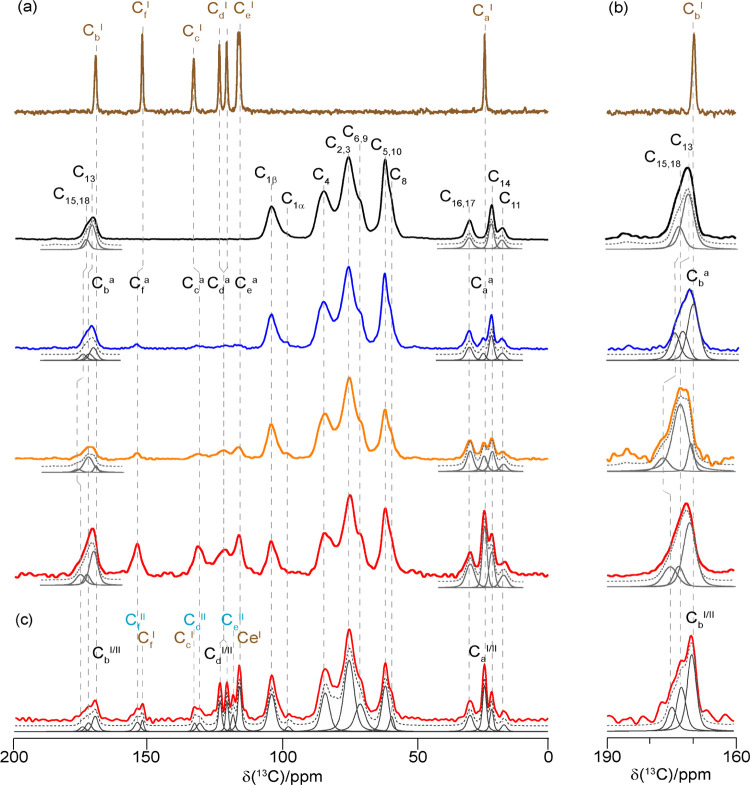
(a) ^13^C CP MAS spectra of crystalline acetaminophen form I (brown), HPMC-AS (black),^69^ 10 wt % (dark blue), 20 wt % (orange), and 40 wt % acetaminophen–HPMC-AS ASDs (red) recorded at less than 1 day at RT/ambient RH. (b) Magnified view of the 190–160 ppm (carbonyl region) of all of the spectra. (c) ^13^C CP spectrum of the 40 wt % dispersion toward recrystallization after 1 week at RT/ambient RH. A magnified view of the aromatic region of this spectrum is given in Figure SI-3. For spectral identification, simulated spectra (dashed gray lines) and spectral deconvolution (gray lines) are also shown. The notations “I” and “II” indicate the characteristic resonances for the acetaminophen forms I and II, respectively, while the notation “a” indicates resonance that can be attributed to amorphous acetaminophen.

Figure 4a compares the ^13^C CP MAS spectra of acetaminophen form I, HPMC-AS polymer, 10, 20, and 40 wt % ASDs. The spectrum of acetaminophen form I presents resonances at around 170 ppm for the carbonyl (C_b_^I^), 152–116 ppm for the aromatic carbons (C_f_^I^, C_c_^I^, C_d_^I^, C_e_^I^), and 24 ppm for the methyl carbon (C_a_^I^) (see Figure 1a and Table SI-2) (“I” indicates characteristic resonances for the acetaminophen form I) based on the previous literature.^68^ The four peaks in the region 105–60 ppm in the spectrum of the HPMC-AS polymer (Figure 1b) can be attributed to the anomeric C_1_, C_4_, C_2,3_, and C_5,10_ carbons, while the shoulders at around 70 and 58 ppm correspond to C_6,9_ (CH_2_s) and C_8_ (methoxy group), and the three peaks in the aliphatic region to C_16,17_ (*C*H_2_ of the S group), C_14_ (*C*H_3_ of the A group), and C_11_ (methyl group of the P moiety). Deconvolution in the carbonyl region of the HPMC-AS polymer reveals two signals assigned to C_15,18_ (most shifted peak, S substituent the *C*Os) and C_13_ (A’s *C*O).^69^ The knowledge of the ^13^C assignment of both the drug and HPMC-AS polymer plays an important role in the identification of drug–polymer interactions in ASDs as this is largely based on the change in chemical shifts.^4,43^ The ^13^C assignments for the ^13^C CP MAS NMR spectra of all ASDs, recorded at less than 1 day at RT/ambient RH, are based on the known spectra of HPMC-AS^69^ and acetaminophen form I and II^68^ (Table SI-2). In the spectra of the ASDs, signals assignable to the amorphous acetaminophen generally appear broader than in the crystalline form as expected from amorphization as the loss of crystallinity brings of a range of chemical environments present that are randomly distributed in the sample resulting in severe inhomogeneous line broadening.^43^ The decreased resolution is evident from the absence of split signals of the aromatic carbons of acetaminophen (C_d_^I^ and C_e_^I^), due to the lack of crystal packing, indicating the presence of amorphous acetaminophen. This is further confirmed by a significant shortening of ^13^C *T*_1_ values by up to 2 orders of magnitude from acetaminophen form I to the amorphous acetaminophen in the ASD (Table SI-3). Meanwhile, the ^13^C *T*_1_ values for HPMC-AS in the ASDs are slightly increased, presumably indicating an increase in rigidity when formulated and suggesting its co-binding in API–polymer interactions (see below). In addition, ^13^C NMR signals for C_d_^I^/C_e_^I^ and quaternary carbons C_f_^a^ show a small difference in chemical shifts of 2–3 ppm vs acetaminophen form I (Table 1). This suggests a structural change in the amorphous systems^43,45,70^ attributed to crystalline API conversion to its amorphous form^71^ and results from the absence of long-range 3D interactions (e.g., hydrogen bonding, π–π interactions) in the crystalline sample, resulting in variation of local electronic environments.

**Table 1:** Selected Significant Changes in ^13^C Chemical Shifts^a^^,^^b^

signal	acetaminophen form I	HPMC-AS	10 wt % ASD	20 wt % ASD	40 wt % ASD	recrystallized 40 wt % ASD
C_15,18_	n.a.	174	174	177	176	174.9
C_13_	n.a.	171	172	173	173	172.4
C_b_	169.7	n.a.	171	171	171	169.8^(I/II)^
C_f_	152.2	n.a.	154	154	154	153.8^(II)^, 152.2^(I)^
C_c_	132.9	n.a.	131	131	131	132.9^(I)^, 130.76^(II)^
C_d_	123.3, 120.5	n.a.	121	121	121	123.3^(I)^, 120.5^(I/II)^
C_e_	116.3, 115.6	n.a.	116	116	116	118.3^(II)^, 115.7^(I)^

a A comprehensive list of ^13^C chemical shifts is given in Table SI-2.

b Values are given in ppm. The ^13^C chemical shifts of all assigned resonances are quoted within an accuracy of ±1 ppm due to the broad line widths associated with amorphous samples, except for the crystalline species where they are quoted at ±0.5 ppm. “I” and “II” indicate resonances belonging to acetaminophen form I and II, respectively.

Furthermore, and more importantly, the carbonyl carbons of the A and S units (C_15,18_ and C_13_) in the ASDs appear to be sensitive to the amount of amorphous acetaminophen in the ASDs as a slight change in chemical shifts vs HPMC-AS to a higher frequency is observed (Figure 4b and Table 1), as shown by the deconvoluted signals for the 190–160 ppm region of the spectra that assumed the presence of three carbonyl signals C_b_, C_13,15_, and C_18_ “three signals model” and supported by residual spectra (Figure SI-4). These shifts are ascribed to API–polymer intramolecular interaction in ASDs and detect molecular association via H-bonding in dispersions,^31,72,73^ as previously observed in the posaconazole (POSA) and HPMC-AS ASD.^44^

**Figure 5:**
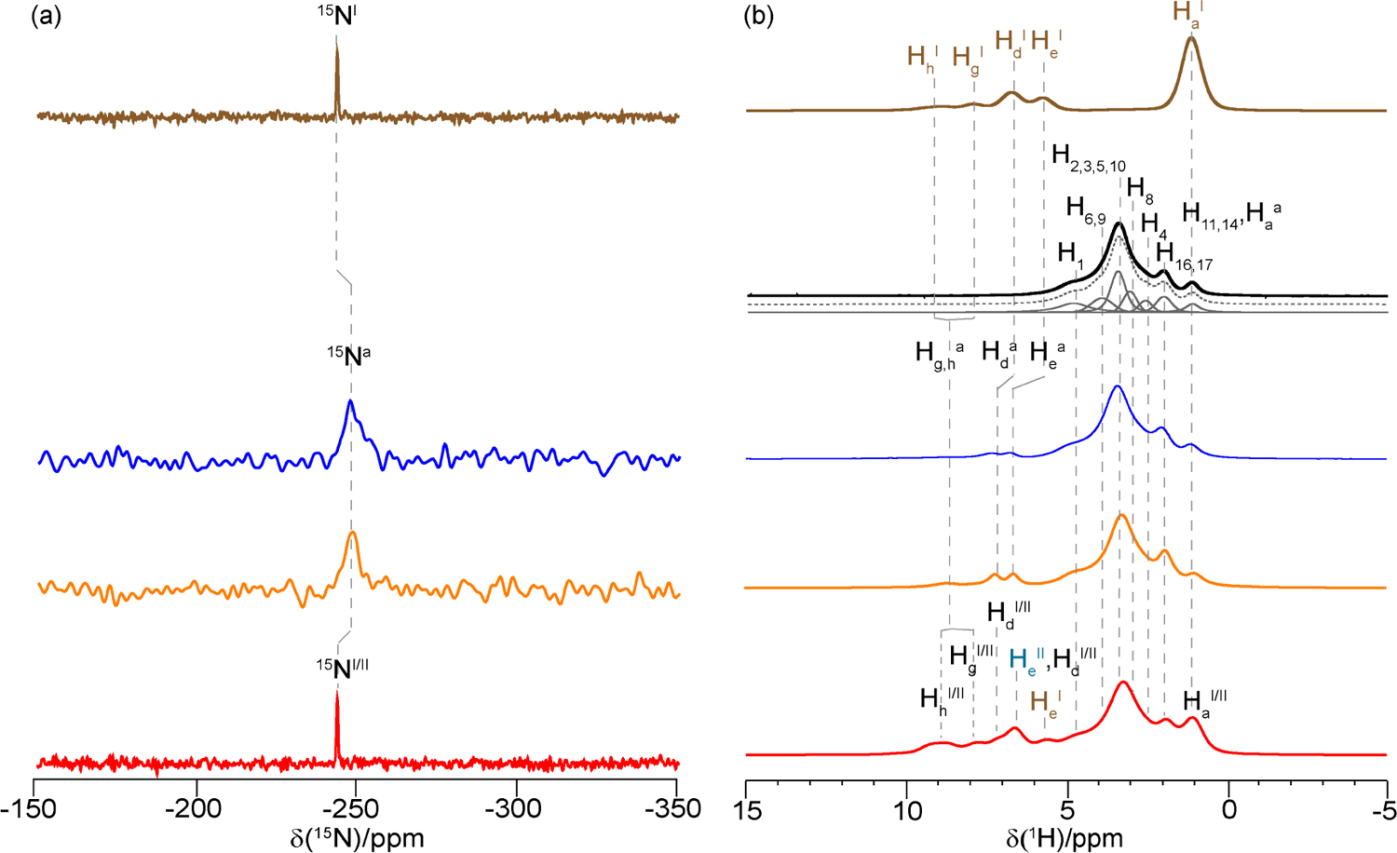
(a) ^15^N CP MAS spectra of crystalline acetaminophen form I (brown), 10 wt % (dark blue), 20 wt % (orange), and 40 wt % (red) acetaminophen–HPMC-AS ASDs. (b) Quantitative ^1^H spectra of crystalline acetaminophen form I (brown), HPMC-AS (black), 10 wt % (dark blue), 20 wt % (orange), and 40 wt % (red) acetaminophen HPMC-AS ASDs after recrystallization. ^1^H signal assignment is based on the ^13^C and ^15^N CP HETCOR experiments in Figures 6a and SI-5, respectively. Magnified views of both ^15^N CP and ^1^H spectra can be found in Figures SI-6 and SI-7, respectively.

The ^13^C CP MAS NMR spectrum of the 40 wt % ASD was also recorded after 1 week under ambient conditions (Figure 4c) and shows significant differences with the one obtained at less than 1 day at RT/ambient RH. The spectrum exhibits a number of additional and sharper peaks as well as a lengthening of the ^1^H *T*_1_’s (Table SI-4), indicating the presence of crystalline acetaminophen arising from fast recrystallization from the ASDs. The resonances observed in Figure 4c (a magnified view of the aromatic region of this spectrum is given in Figure SI-3) indicate the presence of signals that can be attributed to both acetaminophen form I (Figure 4a, brown) and II^68^ as anticipated from the PXRD data (Figure 2). The presence of signals attributable to the two polymorphs of acetaminophen in 40 wt % ASD strongly indicates the instability of this dispersion toward recrystallization and could be reasonably explained by the lack of any interaction between acetaminophen and HPMC-AS, as predicted by the significant negative deviation from the GT model (Figure 1). Figure 5a compares the ^15^N CP MAS NMR spectra of acetaminophen form I, 10 and 20 wt % amorphous acetaminophen in HPMC-AS solid dispersion, which show one signal assignable to the acetaminophen NH amide group (Figure 1a). This peak resonates at −243 ppm and is fairly narrow (full-width-at-half maximum, FWHM of 26 Hz), which is consistent with the literature data for acetaminophen form I,^68^ while the signal appears at −247 ppm for both 10 and 20 wt % ASDs and is significantly broader (FWHM of 240–260 Hz). The change in the ^15^N chemical shift and broadening of the ^15^N spectra observed between crystalline and amorphous species suggests a different hydrogen-bonding network and intramolecular interactions (Table 2).^42,45,74^ The ^15^N CP spectrum of the 40 wt % ASD shows a single resonance at −243 ppm (FWHM of 24 Hz) at the same chemical shift for crystalline form I and likely arises from acetaminophen that underwent recrystallization during data acquisition. Although acetaminophen forms I and II in the 40 wt % ASD have been observed in both PXRD and ^13^C NMR data, the two expected ^15^N signals are not resolved at 9.4 T, likely due to their very similar chemical shift values only separated by 0.4 ppm.^68^

**Table 2:** Experimental ^15^N Isotropic Chemical Shifts δ_iso_(^15^N), ^14^N Shifts δ_iso_(^14^N), ^14^N Quadrupolar-Induced Shifts δ_iso_^Q^(^14^N), and Quadrupolar Products *P*_Q_

sample	δ_iso_(^15^N)^a^	δ_iso_(^14^N)^a^	δ_iso_^Q^(^14^N)^a^	*P*_Q_^b^
acetaminophen form I^51^^c^	–244	–125	119	2.5
10 wt % ASD^c^	–247	–75	172	2.9
20 wt % ASD^d^	–247	–80	167	2.9
recrystallized 40 wt % ASD^c^	–243	–67	176	2.9

a Shifts are given in ppm. δ_iso_(^15^N) values are obtained from the peak positions in the ^15^N CP MAS spectra (with an associated error of ±1 ppm), while δ_iso_(^14^N) values represent the center of gravity of the ^14^N line shape extracted from the ^14^N–^1^H HMQC spectra (with an associated error of ±5 ppm). Magnetic field-dependent ^14^N shifts quoted in the table are given for 20 T.

b *P*_Q_ values are given in MHz, with an estimated error of ±0.1 MHz, and obtained from eq 5.

c Experimental data obtained at 20 T.

d Experimental data obtained at 18.8 T (Table SI-5) and quoted at 20 T.

The ^1^H MAS NMR spectrum (Figure 5b) of acetaminophen form I, obtained under a high magnetic field (>18.8 T) and very fast MAS frequency (>50 kHz), shows fairly resolved resonances at around 9.0, 7.9, 6.7, 5.7, and 1.1 ppm assigned to −NH and −OH groups, aromatic protons (H_d_^I^ and H_e_^I^), and methyl groups, respectively. In the HPMC-AS spectrum, the three main peaks and the peak at 1.1 ppm can be assigned to H_1_, H_2,3,5,10_, H_16,17_, and H_11,14_, respectively, while spectral deconvolution reveals additional signals at 3.9, 3.0, and 2.5 ppm that are assigned to H_6,9_, H_8_, and H_4_, respectively. Due to possible exchange phenomena, the H_7_, −O*H* (hydroxypropyl substituent group), and the −CO_2_*H* (succinoyl moiety) proton signals (Figure 1) are not observed in the 1D and ^13^C CP HETCOR spectra.

**Figure 6:**
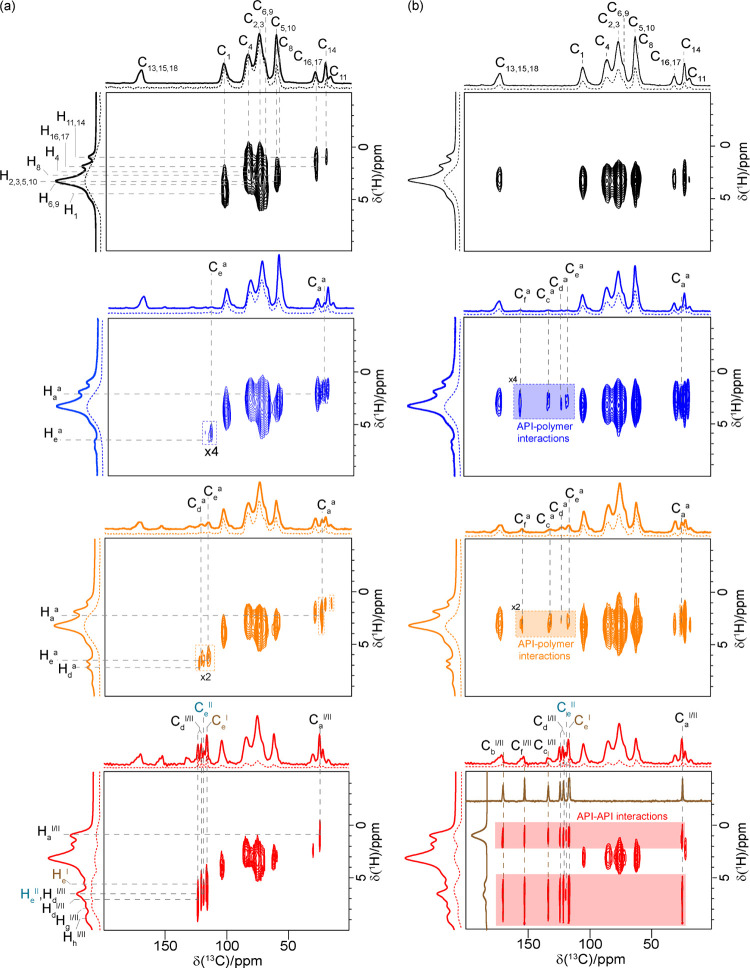
^13^C CP HETCOR spectra of HPMC-AS (black), 10 wt % (dark blue), 20 wt % (orange), and 40 wt % (red) acetaminophen HPMC-AS ASD recorded with a contact time of (a) 50 μs and (b) 2 ms. Correlations were used for ^1^H spectral assignments. Top: ^13^C CP MAS spectra at a contact time of 2 ms. Left: ^1^H MAS NMR spectra. Internal projections are shown in dotted lines. The ^13^C CP MAS NMR spectrum of acetaminophen form I is also given (brown). In panel (a), the dashed lines are used to highlight correlations used for ^1^H assignment. For clarity, the correlation peaks for the polymer are only highlighted for the polymer’s HETCOR in panel ((a), black). In panel (b), the dashed lines denote the carbon signal involved in API–polymer interaction or API–API interaction for the 40 wt % dispersion, while the shaded sections in the spectra mark the cross-correlation peaks that show the API–polymer interactions. Figure SI-8 in the Supporting Information shows a magnified view of the ^13^C region at around 110–200 ppm, highlighting the API–polymer interactions.

The ^1^H spectra of HPMC-AS and all three ASDs (Figure 5b) are assigned from correlations observed in the ^13^C CP HETCOR spectra recorded at a short contact time (Figure 6a), ^15^N CP HETCOR spectrum of the 20 wt % (Figure SI-5), and known ^1^H chemical shifts. The ^1^H spectra of the ASDs (Figure 5b) show a cluster of signals around 5 and 1 ppm, corresponding to HPMC-AS, as well as additional resonances for the acetaminophen. As summarized in Table 3, in the 10 and 20 wt % ASDs, the aromatic proton signals (H_d_^a^ and H_e_^a^) are deshielded with respect to the crystalline counterpart, the small difference observed being typical of amorphization processes.^71^ Finally, the H_g_^a^ and H_h_^a^ signals merged into a single broad signal centered at 8.5 ppm, which correlates strongly with the ^15^N signal as revealed by the ^15^N CP HETCOR spectrum (Figure SI-5), potentially confirming the absence of deprotonation. It has been demonstrated that the deprotonation effect, promoted by the solvent during the spray dry process, might impact API–polymer interactions.^45^ It is well known that evaluation of the length scale of spin diffusion allows the degree of mixture miscibility to be determined by recording the ^1^H relaxation times of all components.^75−78^^1^H *T*_1_ and *T*_1ρ_ values have therefore been measured for the API and polymer in the ASD (Tables SI-4 and SI-6) and revealed that, for the 20 wt % formulation, they are similar (e.g., ^1^H *T*_1_ values for H_d_^a^ and H_1_ are 1.7 ± 0.2 and 1.9 ± 0.3 s, respectively, and ^1^H *T*_1ρ_ (H_d_^a^) = 3.8 ± 1.1 ms ≈ ^1^H *T*_1ρ_ (H_1_) = 4.4 ± 0.3 ms at a spin-lock frequency of 40 kHz), indicating that in the 2–5 nm length scale,^79^ there is miscibility in the acetaminophen–HPMC-AS ASD. In contrast, significantly different ^1^H *T*_1_ and *T*_1ρ_ values are obtained for the recrystallized 40 wt % dispersion (Table SI-6), suggesting that phase separation phenomena occur in a domain size larger than 20–50 nm.^79^

**Table 3:** Significant Changes Observed in the ^1^H Chemical Shifts for Selected Protons^a^

signal	acetaminophen form I	10 wt % ASD	20 wt % ASD	recrystallized 40 wt % ASD
H_h_	9.0	8.5	8.5	9.0^(I/II)^
H_g_	7.9	8.5	8.5	7.9^(I/II)^
H_d_	6.7	7.4	7.4	7.2^(I/II)^, 5.7^(I/II)^
H_e_	5.7	6.8	6.8	6.8^(II)^, 5.7^(I)^

a Values are given in ppm. A comprehensive list of ^1^H chemical shifts can be found in Table SI-7. The associated error with the chemical shift values is ±0.2 ppm.

Through-space ^13^C CP HETCOR experiments recorded at a longer contact time (in the range of ms) allow observation of acetaminophen–polymer interactions with correlation signals providing direct evidence of intermolecular drug polymer interactions. The corresponding ^13^C CP HETCOR spectra of both 10 and 20 wt % ASD identified correlation signals between peaks in ^13^C at 120–150 ppm corresponding to acetaminophen with ^1^H at 3 ppm (shaded signals in Figure 6b). These spatial correlations, detected via the strong ^13^C–^1^H heteronuclear dipolar coupling due to the rigid protons on the cellulose ring, cannot be ascribed to intramolecular correlations within acetaminophen due to the absence of ^1^H signals at this shift (Figure 5b) but rather an intermolecular acetaminophen–HPMC-AS interaction involving the aromatic carbons of acetaminophen with the backbone cellulose ring’s protons of the polymer.

In sharp contrast, the ^13^C CP HETCOR spectrum of 40 wt % ASD identifies correlated signals corresponding to crystalline acetaminophen interacting with itself, as shown by the shaded signals in Figure 6b (no 2D correlation is observed for the broader shoulders of the 1D spectrum likely due to the poor signal-to-noise ratio in the HETCOR of the minor amorphous acetaminophen species). This suggests the absence of acetaminophen–HPMC-AS intramolecular interaction and indicates a two-phase immiscible system in which API–API interactions dominate, in good agreement with GT predictions, the presence of acetaminophen recrystallization and validating ^1^H relaxation data (see above), thereby confirming the instability of this ASD at the atomic level.

**Figure 7:**
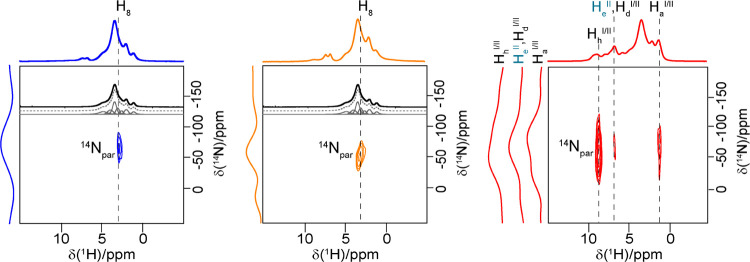
^14^N–^1^H HMQC experiments of 10 wt % (dark blue), 20 wt % (orange), and 40 wt % (red) acetaminophen HPMC-AS ASDs obtained at a MAS frequency of 60 kHz. Data for 10/40% and 20 wt % were collected at 20 and 18.8 T, respectively. Spectra were recorded with recoupling times of 133.6 μs (10 wt % ASD, 8 rotor periods), 66.8 μs (20 wt % ASD, 4 rotor periods), and 801.6 μs (40 wt % ASD, 48 rotor periods). The deconvoluted ^1^H spectra of HPMC-AS under the same condition are also given in black. Spectra on the left of the 2D HMQC are the ^14^N slices extracted at the indicated ^1^H chemical shifts in dashed black lines.

^14^N–^1^H HMQC experiments were then deployed under optima conditions of high magnetic field and very fast MAS frequency to establish the involvement of the amide nitrogen in the intermolecular interactions in these ASDs. ^14^N is a high abundance spin (99.6%) but due to its low gyromagnetic ratio (1.93 × 10^7^ rad T^–1^ s^–1^) and spin quantum number *I* = 1, ^14^N has low sensitivity and exhibits quadrupole interaction, leading to a significant signal broadening. For these reasons, the direct detection of the ^14^N signal in the solid-state represents a challenge. The development of indirectly detected ^14^N via ^1^H as for example via 2D ^14^N–^1^H HMQC experiments at high magnetic field and very fast MAS frequency has enabled the solving of this challenge, establishing this approach as a promising methodology for identifying H-bonding between components in pharmaceutical systems.^51,52,80^ The corresponding ^14^N–^1^H HMQC experiments for the 10 and 20 wt % ASDs (Figure 7) identify the presence of correlation between the acetaminophen ^14^N^a^ signal with the −OCH_3_ methoxy group (H_8_) of the polymer at 3 ppm and highlight H-bonding between this amide donor and oxygen acceptor. In the spectra, no correlation between the NH group of paracetamol and the protons of the substituent groups P, A, and S (Figure 1) was identified, thus excluding the involvement of these groups in the formation of the H-bond between API and the polymer.

Importantly, this ^14^N signal correlating with H_8_ does not correspond to the same proton (H_h_^a^) identified via ^15^N CP HECTOR that established the NH correlation within acetaminophen (Figure SI-5) and suggests longer-range interactions. We note that this interaction for the 10 and 20 wt % amorphous dispersions was identified using short recoupling times of 133.6 and 66.8 μs, respectively, suggesting a closer contact between acetaminophen and HPMC-AS in those systems than in crystalline acetaminophen, which is consistent with the previous work in the amorphous formulation.^52^ It is proposed that this H-bonding interaction is dominant to stabilize acetaminophen in its amorphous form in these ASDs.

**Figure 8:**
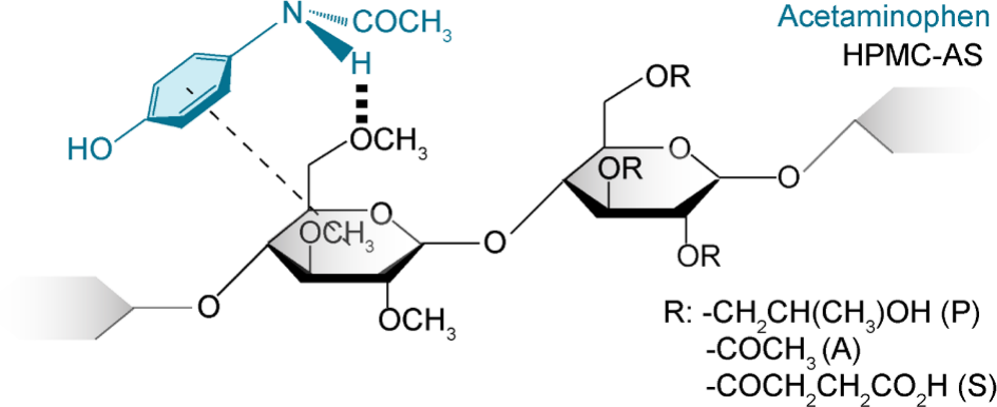
Schematic representation of the interactions that have been experimentally identified in this work. For dispersions with a drug loading of <20 wt %, spatial proximity (---) and H-bond (≣) were identified between the API and the polymer. The acetaminophen and HPMC-AS molecules are given in light blue and black, respectively.

In contrast to the 10 and 20 wt % ASDs, the ^14^N–^1^H HMQC spectrum for the 40 wt % ASD clearly exhibits correlations between the ^14^N and ^1^H (H_h_^I/II^, H_e_^II^/H_d_^I/II^, and H_a_^I/II^) signals within acetaminophen and no correlation to the HPMC-AS polymer, confirming the absence of acetaminophen–HPMC-AS interactions at this high drug loading. The API–API H-bonding interaction was found at significantly longer recoupling times, reasonably indicating a longer distance between the packed acetaminophen molecules in the crystal structure compared to the API–polymer distance in amorphous systems, as illustrated previously.^50^ For both the 10 and 20 wt % amorphous dispersions, the correlation signals in the ^13^C CP HETCOR experiments carried out at long contact times (Figure 6b) and ^14^N–^1^H HMQC spectra at short recoupling times (Figure 7) highlight intermolecular amorphous drug–polymer H-bonding interactions (Figure 8).

The experimental ^14^N shifts for the observed signals in Figure 7 and the ^15^N isotropic chemical shifts obtained in the ^15^N CP experiments (Figure 5a) for the 10 and 20 wt % ASDs are listed in Table 2. The differences in shifts between ^14^N and ^15^N are due to the ^14^N isotropic second-order quadrupolar shift, which is given by eq 3
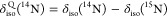
3and allows the determination of the quadrupolar product *P*_Q_ from eq 4(50)
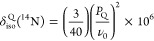
4where ν_0_ is the ^14^N Larmor frequency. *P*_Q_ depends on the quadrupolar coupling constant *C*_Q_ and asymmetry parameter η_Q_, as expressed by eq 5
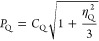
5

A significant difference of around 180–190 ppm between ^15^N isotropic chemical shift and ^14^N shift is observed (Table 2) in the acetaminophen HPMC-AS ASD. This is attributed to the isotropic second-order quadrupolar shift being sensitive to the presence of the H-bond, as previously observed in the 50 wt % acetaminophen–PVP solid dispersion that extracted a δ_iso_^Q^(^14^N) value of around 184 ppm.^51^ This data further supports the presence of acetaminophen HPMC-AS H-bond in the dispersions with drug loading <20% wt and acetaminophen–acetaminophen H-bond interaction in the recrystallized 40% ASD.

These are stabilizing interactions that can be imputed in the understanding of the stability of the amorphous acetaminophen–HPMC-AS solid dispersions. Interestingly, the main stabilizing interaction that has been identified in this work is H-bonding between the acetaminophen’s amide group with the OCH_3_ proton (H_8_) of the HPMC-AS methyl substituent (M), likely due to the small steric hindrance of this substituent vs the others (Figure 1). This is an unexpected finding given that the acetyl and succinoyl groups in HPMC-AS have been previously suggested to be responsible for the formation of API–polymer H-bonding and contribute to the formation of stabilizing interactions.^42^

## 4 Conclusions

Molecular interactions in acetaminophen–HPMC-AS solid dispersion at 10, 20, and 40 wt % drug loadings were identified by combining time-dependent PXRD with multidimensional multinuclear NMR experiments. The presence of chemical shift differences in 1D ^1^H, ^13^C, and ^15^N CP MAS NMR spectra between crystalline and amorphous acetaminophen suggests a strong structural perturbation in the amorphous species and can be potentially rationalized by the presence of H-bonding interactions between acetaminophen and the polymer. ^13^C CP HETCOR exploiting strong ^13^C–^1^H dipolar coupling highlighted spatial interaction between the acetaminophen’s aromatic protons with the polymer’s cellulose ring protons in the 10 and 20 wt % ASDs. This interaction was further unequivocally confirmed by ^14^N–^1^H HMQC experiments that identify H-bond interactions between the NH of acetaminophen and the OCH_3_ proton of the HPMC-AS methyl substituent. The presence of this type of drug/polymer interaction in amorphous systems is of crucial importance as it stabilizes the amorphous dispersions. No acetaminophen–HPMC-AS interactions were found in the 40 wt % dispersion, further validated from ^1^H relaxation data, indicating the instability of this system and its tendency to recrystallize on a short timescale.

## Supporting Information Available

Acetaminophen and HPMC-AS chemical structures; magnified view of the 190–160 ppm ^13^C region of the ^13^C CP spectrum of the recrystallized 40% ASD; residual ^13^C spectrum between experimental and simulated spectra; ^13^C CP and HETCOR spectra of the 20 wt % ASD after 1 year at RT/ambient RH; ^15^N CP HETCOR of the 20 wt % ASD; magnified views of ^1^H, ^13^C CP, ^15^N CP, and ^13^C CP HETCOR spectra; summary of Tg values; tables of ^1^H and ^13^C chemical shifts and *T*_1_ relaxation times, ^1^H *T*_1ρ_ relaxation times, experimental ^15^N isotropic chemical shifts δ_iso_(^15^N), ^14^N shifts δ_iso_(^14^N), ^14^N quadrupolar-induced shifts δ_iso_^Q^(^14^N), and quadrupolar products *P*_Q_ for the 20 wt % ASD recorded at 18.8 T (PDF)
