# Supplementary material for: Drug–Polymer Interactions in Acetaminophen/Hydroxypropylmethylcellulose Acetyl Succinate Amorphous Solid Dispersions Revealed by Multidimensional Multinuclear Solid-State NMR Spectroscopy
Source: Mol Pharm. 2021 Aug 10;18(9):3519–31. doi: 10.1021/acs.molpharmaceut.1c00427 (PMC8424625; doi:10.1021/acs.molpharmaceut.1c00427)
Supplement: Supplementary file 1 — mp1c00427_si_001.pdf [file mp1c00427_si_001.pdf]

Supporting Information For

Drug-polymer interactions in acetaminophen / hydroxypropylmethylcellulose acetyl succinate amorphous solid dispersions revealed by multidimensional multinuclear solid-state NMR spectroscopy

Andrea Pugliese,[a] Michael Toresco,[b] Daniel McNamara,[c] Dinu Iuga,[d] Anuji Abraham,[c] Michael Tobyn,[e] Lucy E. Hawarden,[e] and Frédéric Blanc*,[a,f]

[a] Department of Chemistry, University of Liverpool, Crown Street, Liverpool L69 7ZD, UK.

[b] Rowan College of Engineering, Chemical Engineering Department, Rowan University, Mullica Hill Road, Glassboro, New Jersey 08028, US

[c] Drug Product Development, Bristol-Myers Squibb, One Squibb Drive, New Brunswick, New Jersey 08903, US.

[d] Department of Physics, University of Warwick, Gibbet Hill Road, Coventry CV4 7AL, UK.

[e] Drug Product Development, Reeds Lane, Moreton CH46 1QW, UK.

[f] Stephenson Institute for Renewable Energy, University of Liverpool, Peach Street, Liverpool L69 7ZF, UK

*To whom correspondence should be addressed to. Email: frederic.blanc@liverpool.ac.uk

CONTENTS

Figure SI-1. Acetaminophen and HPMC-AS chemical structures ……………………………………………....Pag. 2

Figure SI-2. ^{13}C CP HETCOR spectra for the 20% wt. acetaminophen HPMC-AS solid dispersion after 1 year at room temperature (RT) and ambient room humidity (RH)……………………………………………………………...2

Figure SI-3. Magnified view of the 160–110 ppm ^{13}C region for the ^{13}C CP spectrum of 40% wt. acetaminophen in HPMC-AS solid dispersion after 1 week at RT and ambient RH…………………………………………………..………3

Figure SI-4. Residual ^{13}C spectrum between experimental and simulated spectra …………………………..……4

Figure SI-5. ^{15}N CP HETCOR of the 20% acetaminophen HPMC-AS solid dispersion……………………………….5

Figure SI-6. Magnified view of the ^{15}N CP spectra….…………………………………………………………..……..6

Figure SI-7. Magnified view of the quantitative ^{1}H spectra…………………………..……………………………..…...7

Figure SI-8. Magnified view of the 200-110 ppm ^{13}C region of the ^{13}C CP HETCOR spectra………………………….8

Table SI-1. Tg values obtained for the acetaminophen HPMC-AS ASDs ……………………………..…………..…9

Table SI-2. ^{13}C chemical shifts………………………………………………………………………………….....……9

Table SI-3. ^{13}C T_1 relaxation times values measured at 9.4 T………………………………………………………..…10

Table SI-4. ^{1}H T_1 relaxation times values measured at 18.8 T…………………………………………………………10

Table SI-5. Experimental ^{15}N isotropic chemical shifts $\delta_{iso}(^{15}N)$, ^{14}N shifts $\delta_{iso}(^{14}N)$, ^{14}N quadrupolar-induced shifts δ_{iso}^{Q} (^{14}N) and quadrupolar products P_Q for the 20% wt. ASD recorded at 18.8 T…………………...………….…..11

Table SI-6. ^{1}H $T_{1\rho}$ values for the 10% wt., 20% wt. and recrystallised 40% wt. at 9.4 T recorded at two different spin locks amplitude (83, and 40 kHz)……………………………………..………………………..……………......…11

Table SI-7. ^{1}H chemical shifts…………………………………………………………………………………………...12

References……………………………………………………………………………………… ……………...……13

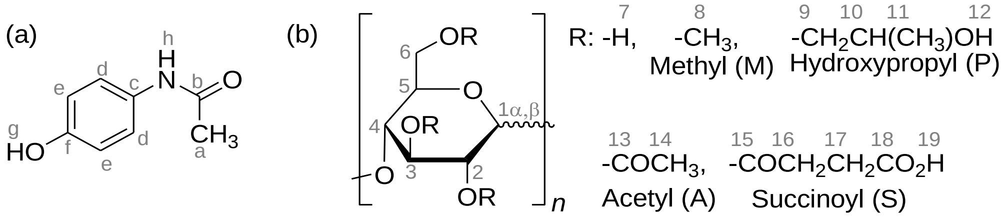

Figure SI-1. (a) Acetaminophen and (b) Hydroxypropylmethylcellulose acetyl succinate (HPMC-AS) polymer chemical structures. The lettering and numbering are used for all NMR spectral assignments throughout.

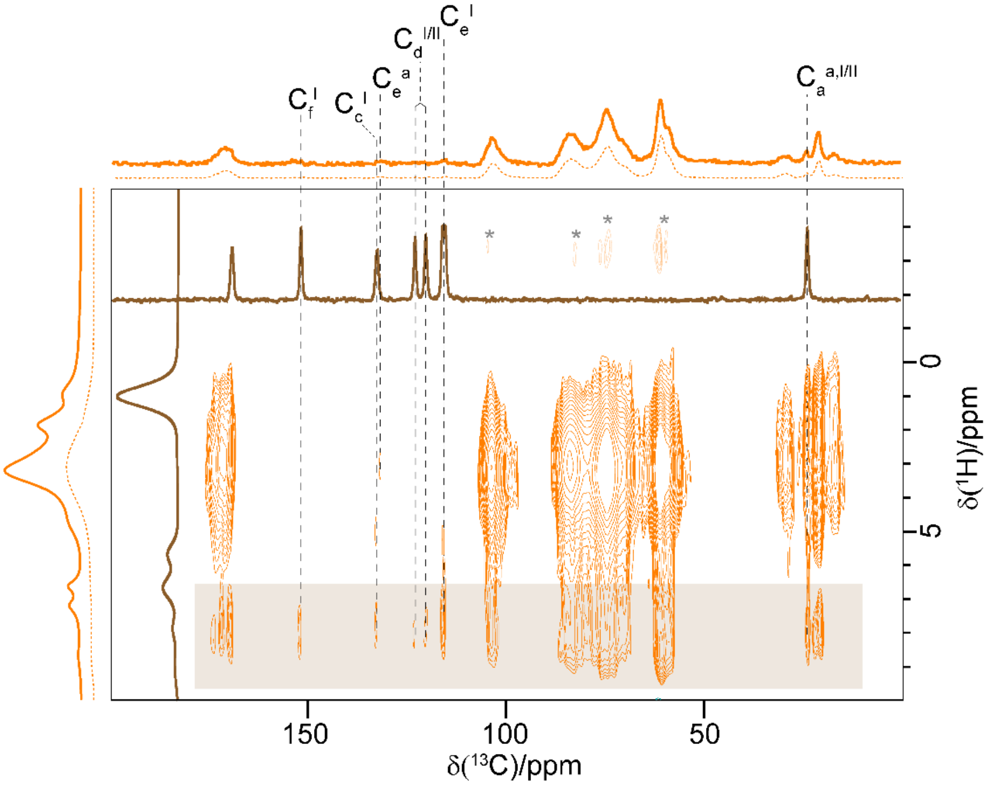

Figure SI-2. ^{13}C CP HETCOR recorded at contact time of 2 ms for the 20% wt. acetaminophen HPMC-AS solid dispersion after 1 year at room temperature (RT) and ambient room humidity (RH). The shaded section denotes drug-drug correlations from presence of acetaminophen form I in the sample, confirming the PXRD data (Figure 2, panel (g)). Top: ^{13}C CP MAS spectrum. Left: ^{1}H MAS NMR spectrum recorded at high magnetic field. The dashed spectra are internal projections. ^{1}H and ^{13}C CP spectra of acetaminophen form I are given in brown. The notation 'I' indicates the characteristic resonances for the acetaminophen form I, while the notation 'a' indicates resonances that can be attributed to amorphous acetaminophen. Asterisks denote speed sidebands.

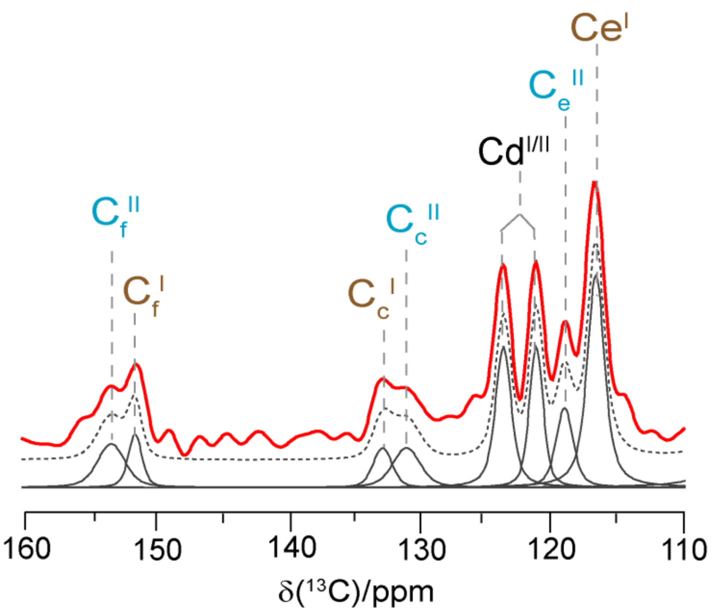

Figure SI-3. Magnified view of the 160–110 ppm ^{13}C region for the ^{13}C CP spectrum of 40% wt. acetaminophen in HPMC-AS solid dispersion after 1 week at RT and ambient RH. Simulated spectra (dashed grey lines) and spectral deconvolution (grey lines) are given and allowed assignment of all the resonances in paracetamol forms I and II based on literature data.[1]

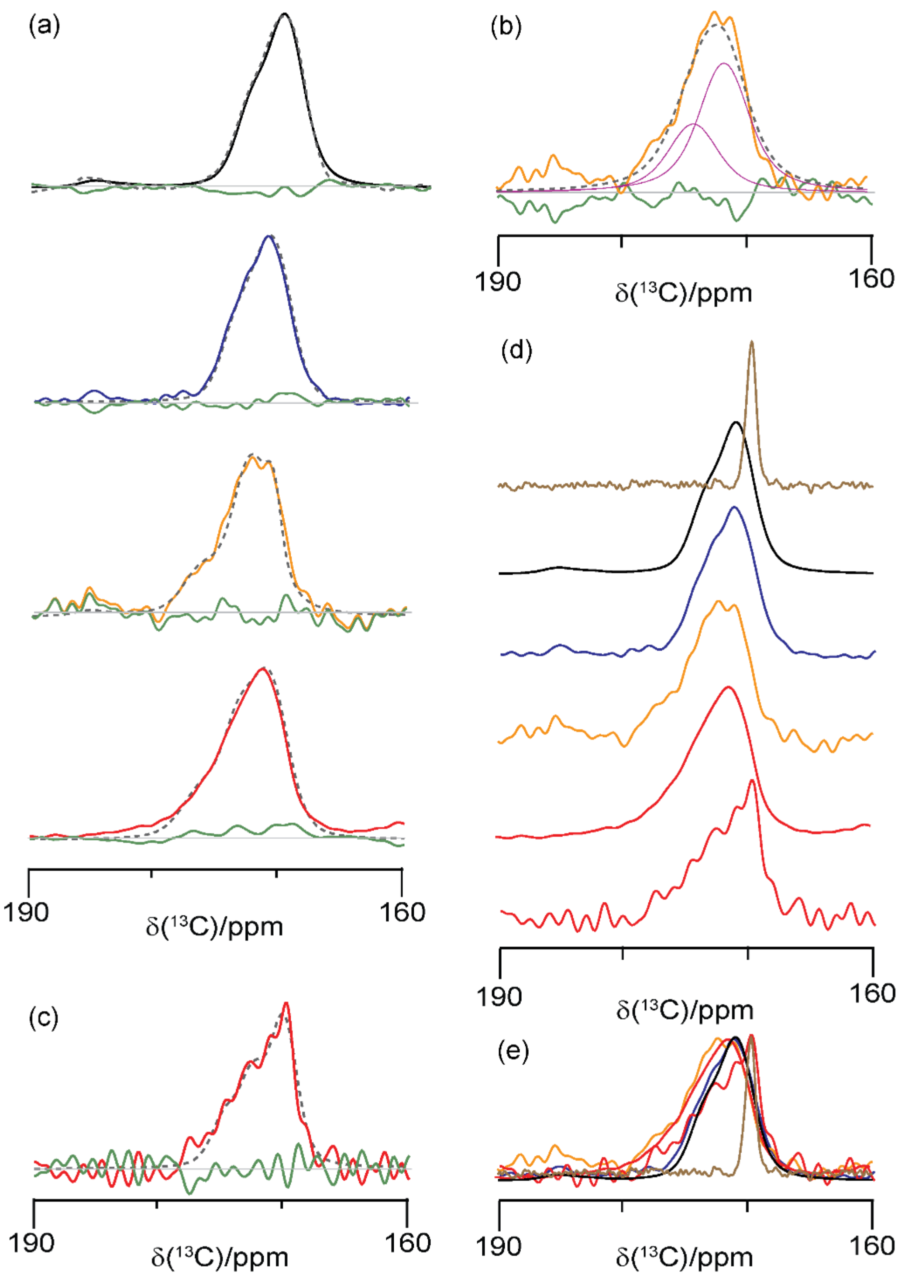

Figure SI-4. (a) Residual ^{13}C spectrum (in green) between experimental and simulated spectra using the "three signals model". Experimental spectra for HPMC-AS, 10% wt., 20% wt., and 40% wt. acetaminophen dispersion are given in black, dark blue, orange, and red, respectively while simulated spectra are provided as dark grey dashed lines. The horizontal light grey lines mark the zero-intensity signal. (b) Residual ^{13}C spectrum (in green) between experimental and simulated spectra for the 20% wt. ASD using two peaks only (in violet) rather than the "three signals model" of (a). (c) Residual ^{13}C spectrum obtained for the 40% wt. dispersion towards recrystallisation. (d) Stacked and (e) Overlaid spectra overview in the 190–160 ppm (carbonyl) region. The crystalline acetaminophen form I ^{13}C CP spectrum is given in brown for comparison.

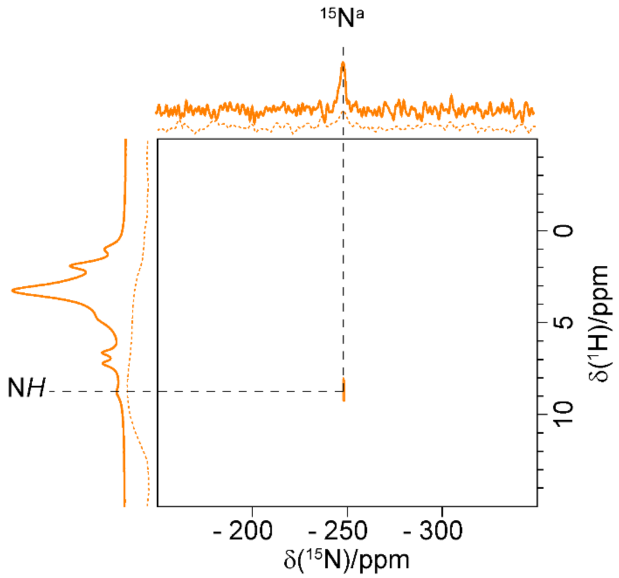

Figure SI-5. ^{15}N CP HETCOR of the 20% acetaminophen HPMC-AS solid dispersion recorded with an optimised contact time of 1 ms. On top: ^{15}N CP spectrum. On the left: ^{1}H spectrum. Dashed spectra are the HETCOR internal projections. The 2D spectra shown only one resonance at $\delta(^{1}H)$ = 8.5 ppm confirming the proposed ^{1}H assignment for acetaminophen's N*H* in amorphous samples. Notation 'a' indicates resonance that can be attributed to amorphous acetaminophen.

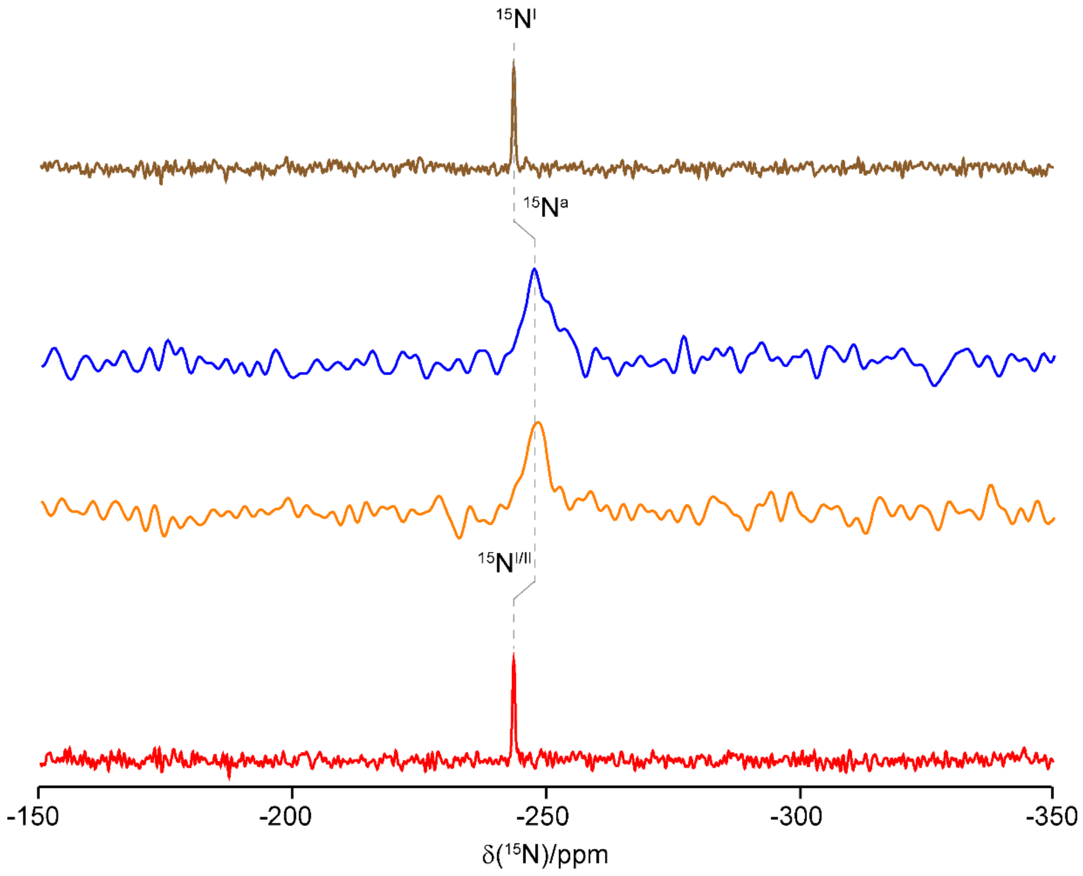

Figure SI-6. Magnified view of the ^{15}N CP spectra. From the top to the bottom: crystalline acetaminophen form I, 10% wt., 20% wt., and recrystallised 40% wt. ASDs.

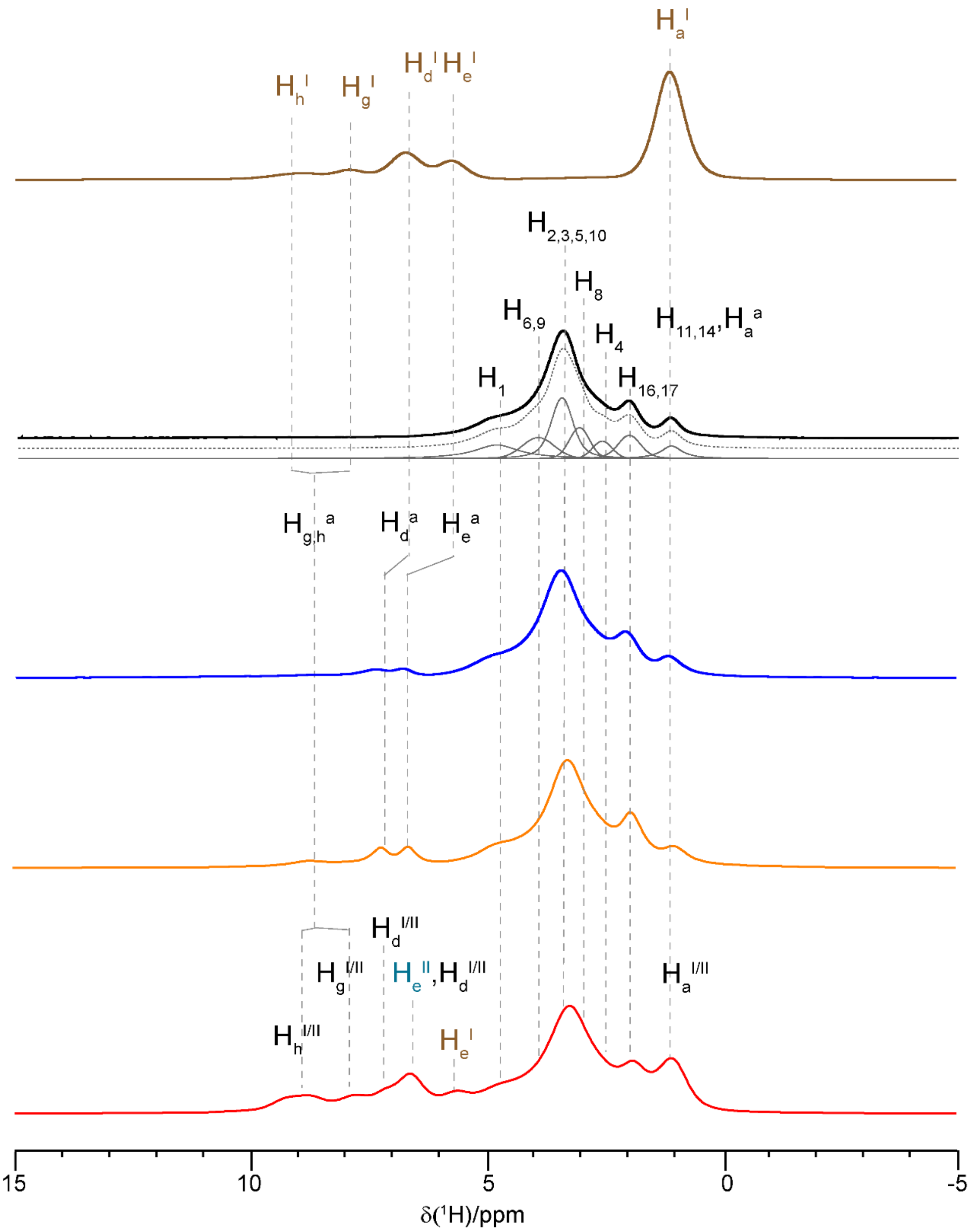

Figure SI-7. Magnified view of the quantitative ^{1}H spectra recorded at high magnetic field and high MAS frequency. From the top to the bottom: crystalline acetaminophen form I, HPMC-AS polymer, 10% wt., 20% wt., and recrystallised 40% wt. ASDs.

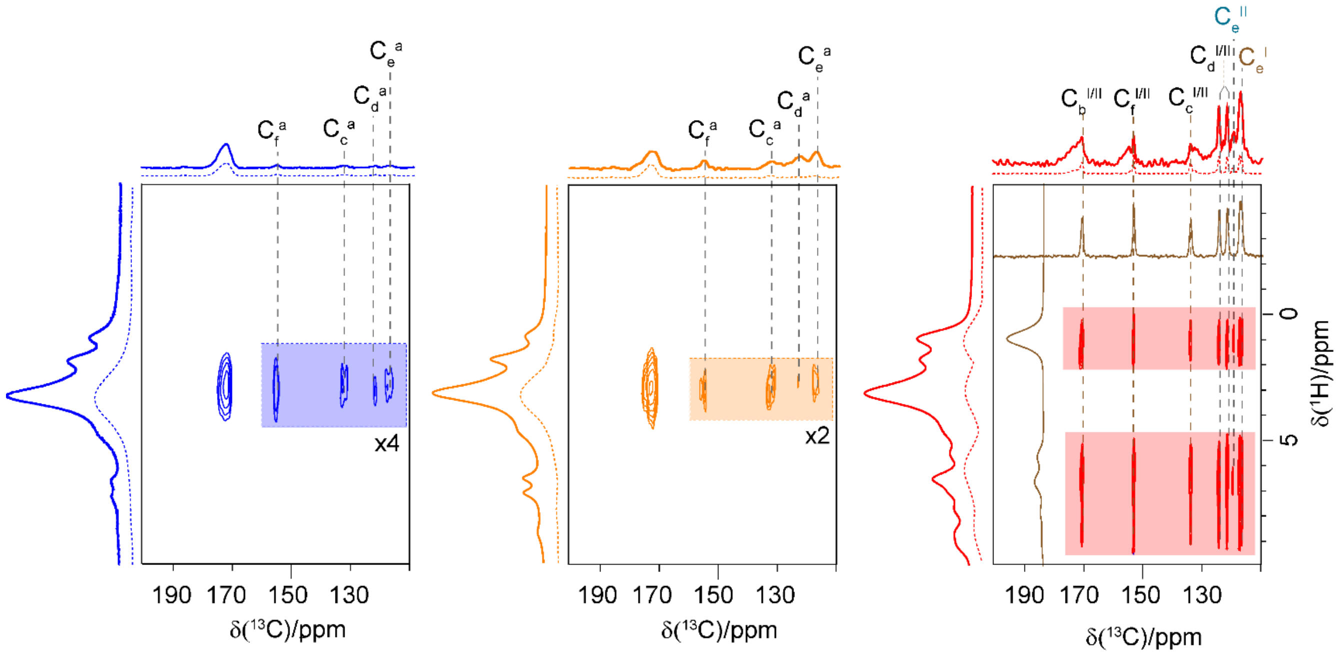

Figure SI-8. Magnified view of the 200-110 ppm ^{13}C region of the ^{13}C CP HETCOR spectra recorded at long contact time (2 ms) showing the API-polymer spatial correlations. The 2D HETCORs for the 10% wt., 20% wt. and 40% wt. dispersions are given in dark blue, yellow and red, respectively. The signals reported in the shaded area are involved in the spatial correlations. The dashed lines denotate the carbon signal involved in API-polymer interaction, or the API-API interaction for the 40% wt. dispersion

Table SI-1. Tg values obtained for the acetaminophen HPMC-AS ASDs.

Acetaminophen (w/w%)	Predicted Tg_{mix} /°C	Experimental Tg /°C	($Tg - Tg_{mix}$) /°C
HPMC-AS	n.a.	120 (ref [2])	n.a.
10	103	76[a]	-27
20	105	74[a]	-31
40	88	45[b]	-43
Acetaminophen	n.a.	25 (ref [2])	n.a.

[a] Experimental values carried out using a standard DSC analysis. [b] Experimental values carried out using mDSC experiment. n.a.: not applicable.

Table SI-2. ^{13}C chemical shifts.[a]

Signal	Acetaminophen form I	HPMC-AS	10% wt. ASD	20% wt. ASD	40% wt. ASD	Recrystallised 40% wt. ASD
$C_{15,18}$	n.a.	174	174	177	176	174.9
C_{13}	n.a.	171	172	173	173	172.4
C_b	169.7	n.a.	171	171	171	169.8 (I/II)
C_f	152.2	n.a.	154	154	154	153.8 (II), 152.2 (I)
C_c	132.9	n.a.	131	131	131	132.9 (I), 130.76 (II)
C_d	123.3, 120.5	n.a.	121	121	121	123.3 (I), 120.5 (I/II)
C_e	116.3, 115.6	n.a.	116	116	116	118.3 (II), 115.7 (I)
$C_{1\alpha}$	n.a.	104	104	104	104	104
$C_{1\beta}$	n.a.	100	100	100	100	100
C_4	n.a.	84	84	84	84	84
$C_{2,3}$	n.a.	75	75	75	75	75
$C_{6,9}$	n.a.	70	70	70	70	70
$C_{5,10}$	n.a.	61	61	61	61	61
C_8	n.a.	58	58	58	58	58
$C_{16,17}$	n.a.	29	29	29	29	29
C_a	23.6	n.a.	24.2	24.1	24.0	23.6 (I/II)
C_{14}	n.a.	21	21	21	21	21.1
C_{11}	n.a.	17	17	17	17	16.9

[a] Values are given in ppm. The ^{13}C chemical shifts of all assigned resonances are quoted within an accuracy of ±1 ppm due to the broad line widths associated with amorphous samples, except for the crystalline species where they are quoted at ± 0.5 ppm. n.a.: not applicable.

Table SI-3. ^{13}C T_1 relaxation times values measured at 9.4 T.[a]

Signal	Acetaminophen form I	HPMC-AS	10% wt. ASD	20% wt. ASD	40% wt. ASD
$C_{15,18}$	n.a.	20 (3)	22 (10)	25 (25)	20 (20)
C_{13}	n.a.	27 (4)	n.d.	34 (3)	67 (48)
C_b	2220 (260)	n.a.	33 (7)	34 (4)	44 (17)
C_f	1050 (200)	n.a.	n.d.	24 (5)	38 (26)
C_c	1450 (280)	n.a.	n.d.	40 (13)	38 (30)
C_d	1440 (230)	n.a.	n.d.	5 (4)	23 (28)
C_e	1480 (250)	n.a.	n.d.	4 (1)	8 (22)
C_1	n.a.	27 (2)	35 (10)	40 (3)	35 (4)
C_4	n.a.	21 (1)	26 (2)	35 (2)	30 (4)
$C_{2,3}$	n.a.	17 (0.7)	22 (0.9)	28 (1)	23 (2)
$C_{6,9}$	n.a.	6 (0.5)	10 (0.9)	11 (1)	7 (2)
$C_{5,10}$	n.a.	7 (0.3)	7 (0.9)	14 (3)	8 (0.8)
C_8	n.a.	4 (0.3)	5 (0.8)	5 (0.4)	5 (0.7)
$C_{16,17}$	n.a.	1 (0.2)	3 (0.9)	3 (0.4)	2 (0.9)
C_a	50 (10)	n.a.	9 (7.3)	11 (1.3)	11 (1)
C_{14}	n.a.	10 (0.6)	11 (1.5)	12 (0.9)	9 (2)
C_{11}	n.a.	1 (0.2)	1 (0.2)	1 (0.2)	1 (0.7)

[a] Values are given in seconds. Numbers in parenthesis indicate the standard error associated with the fit (e.g., ^{13}C T_1 for signal C_b in the acetaminophen form I reads 2220 ± 260 s). In some cases, the signal to noise ratio was too poor to reliably extract T_1 values which have therefore not been determined (n.d.). n.a.: not applicable.

Table SI-4. ^1H T_1 relaxation times values measured at 18.8 T.[a]

Signal	10% wt. ASD	20% wt. ASD	Recrystallised 40% wt. ASD
$H_{g,h}$	0.8 (0.2)	1.2 (0.3)	8.0 (2.0) (g), 5.5 (1.0) (h)
H_d	1.1 (0.2)	1.7 (0.2)	6.5 (0.7) (I), 4.3 (0.6) (II)
H_e	1.1 (0.3)	1.7 (0.3)	4.5 (0.7) (I), 6.5 (0.7) (II)
H_1	1.6 (0.2)	1.9 (0.3)	1.9 (0.2)
$H_{6,9}$	1.9 (0.2)	2.2 (0.3)	2.5 (0.2)
$H_{2,3,5,10}$	2.1 (0.1)	2.5 (0.2)	3.0 (0.1)
H_8	2.0 (0.1)	2.4 (0.2)	3.0 (0.1)
H_4	1.9 (0.1)	2.3 (0.2)	2.8 (0.2)
$H_{16,17}$	2.0 (0.2)	2.3 (0.3)	2.7 (0.1)
H_a	n.d.[b]	n.d. [b]	n.d. [b]
$H_{11,14}$	n.d.[b]	n.d. [b]	n.d. [b]

[a] Values are given in seconds. [b] The ^1H T_1 for H_a and for the succinoyl CH_2s $H_{11,14}$ signals are not determined (n.d.) due to overlapping peaks. The number in parenthesis indicate the standard error associated with the fit.

Table SI-5. Experimental ^{15}N isotropic chemical shifts $\delta_{iso}(^{15}N)$, ^{14}N shifts $\delta_{iso}(^{14}N)$, ^{14}N quadrupolar-induced shifts δ_{iso}^{Q} (^{14}N) and quadrupolar products P_Q for the 20% wt. ASD recorded at 18.8 T.[a]

Sample	$\delta_{iso}(^{15}N)$[a]	$\delta_{iso}(^{14}N)$[a]	$\delta_{iso}^{Q}(^{14}N)$[a]	P_Q[b]
20% wt. ASD	-247	-57	190	2.9

[a] Shifts are given in ppm. $\delta_{iso}(^{15}N)$ values are obtained from the peak positions in the ^{15}N CP MAS spectra (with an associated error of ± 1 ppm) while $\delta_{iso}(^{14}N)$ values represent the centre of gravity of the ^{14}N line shape extract from the ^{14}N-^{1}H HMQC spectra (with an associated error of ± 5 ppm). [b] P_Q values are given in MHz, with an estimated error of ± 0.1 MHz, and obtained from Eq. 5.

Table SI-6. ^{1}H $T_{1\rho}$ values for the 10% wt., 20% wt. and recrystallised 40% wt. at 9.4 T recorded at two different spin locks amplitude (83 and 40 kHz).[a]

Signal	20% wt. dispersion		Recrystallised 40% wt. dispersion	
	83 kHz	40 kHz	83 kHz	40 kHz
H_d^{I}	n.a.	n.a.	> 96[b]	> 50[b]
H_d^{II}	n.a.	n.a.	13.3 (7.6)	> 12[b]
H_d^{a}	7.3 (2.0)	3.8 (1.1)	n.a.	n.a.
H_e^{I}	n.a.	n.a.	7.8 (2.2)	> 11[b]
H_e^{II}	n.a.	n.a.	30.8 (13.1)	3.4 (4.5)[b]
H_e^{a}	8.9 (1.2)	4.1 (0.8)	n.a.	n.a.
H_1	9.2 (0.5)	4.4 (0.3)	5.3 (0.5)	2.4 (0.3)
$H_{2,3}$	9.2 (0.3)	4.2 (0.1)	5.4 (0.3)	2.4 (0.1)
H_4	9.2 (0.3)	4.2 (0.1)	5.3 (0.4)	2.4 (0.2)
$H_{5,10}$	10.5 (0.7)	4.7 (0.1)	5.8 (0.4)	2.6 (0.1)
H_8	10.8 (0.7)	4.4 (0.4)	5.6 (0.7)	2.4 (0.3)
$H_{6,9}$	8.9 (0.3)	4.6 (0.2)	5.6 (0.4)	2.3 (0.2)
$H_{16,17}$	8.6 (0.6)	4.4 (0.6)	4.8 (1.4)	1.9 (1.1)
$H_a^{I/II}$	n.a.	n.a.	6.5 (2.3)	1.5 (1.4)
H_a^{a}	9.6 (0.6)	3.2 (0.4)	n.a.	n.a.
H_{14}	11.1 (1.0)	4.8 (0.5)	5.4 (0.6)	2.5 (0.4)
H_{11}	9.1 (1.7)	5.7 (1.6)	4.5 (1.8)	1.8 (0.5)

[a] Values are given in ms. The number in parenthesis indicate the standard error associated with the fit. [b] Shortest values given as these challenge the detection of the full exponential decays of the signals due to NMR probe limitation on spin-lock pulse duration. n.a.: not applicable.

Table SI-7. ^{1}H chemical shifts.[a]

Signal	Acetaminophen form I	HPMC-AS	10% wt. ASD	20% wt. ASD	Recrystallised 40% wt. ASD
H_h	9.0	n.a.	8.5	8.5	9.0 (I/II)
H_g	7.9	n.a.	8.5	8.5	7.9 (I/II)
H_d	6.7	n.a.	7.4	7.4	7.2 (I/II), 5.7 (I/II)
H_e	5.7	n.a.	6.8	6.8	6.8 (II), 5.7 (I)
H_1	n.a.	4.8	4.8	4.8	4.8
$H_{6,9}$	n.a.	3.9	3.9	3.9	3.9
$H_{2,3,5,10}$	n.a.	3.4	3.4	3.4	3.4
H_8	n.a.	3.0	3.0	3.0	3.0
H_4	n.a.	2.5	2.5	2.5	2.5
$H_{16,17}$	n.a.	2.0	2.0	2.0	2.0
H_{14}	n.a.	1.1	1.1	1.1	1.1
H_a	1.1	n.a.	1.1	1.1	1.1

[a] Values are given in ppm. The associated error with the chemical shift values is ± 0.2 ppm. n.a.: not applicable.

References

(1) Burley, J. C.; Duer, M. J.; Stein, R. S.; Vrcelj, R. M. Enforcing Ostwald's Rule of Stages: Isolation of Paracetamol Forms III and II. *Eur. J. Pharm. Sci.* **2007**, *31*, 271–276.

(2) Lehmkemper, K.; Kyeremateng, S. O.; Bartels, M.; Degenhardt, M.; Sadowski, G. Physical Stability of API/Polymer-Blend Amorphous Solid Dispersions. *Eur. J. Pharm. Biopharm.* **2018**, *124*, 147–157.
